# Proto‐Oncogene *HRAS* Transcript Level and Overall Survival in Stages II and III Colorectal Cancer

**DOI:** 10.1002/cam4.71114

**Published:** 2025-07-31

**Authors:** Donghyun Kim, Saima Sharif, Juan Antonio Raygoza Garay, Avanish S. Bhakta, Patrick M. Boland, Michael J. Cavnar, Michelle L. Churchman, Hassan Hatoum, Lyen C. Huang, Joseph Kim, Richard Kim, Robert W. Lentz, Sarbajit Mukherjee, Mary T. O'Donnell, Benjamin Quartey, Matthew J. Reilley, Robert J. Rounbehler, Bodour Salhia, Bryan P. Schneider, Carlos H. Chan

**Affiliations:** ^1^ Division of Hematology, Oncology and Blood & Marrow Transplantation, Department of Internal Medicine University of Iowa Iowa City Iowa USA; ^2^ Holden Comprehensive Cancer Center, University of Iowa Iowa City Iowa USA; ^3^ Department of Surgery University of Kentucky Lexington Kentucky USA; ^4^ Department of Medical Oncology Rutgers Cancer Institute of New Jersey New Brunswick New Jersey USA; ^5^ Department of Clinical & Life Sciences Aster Insights Hudson Florida USA; ^6^ Section of Hematology‐Oncology, Department of Internal Medicine University of Oklahoma Health Sciences Oklahoma City Oklahoma USA; ^7^ Division of Surgical Oncology, Department of Surgery, Huntsman Cancer Institute University of Utah Salt Lake City Utah USA; ^8^ Department of Gastrointestinal Oncology Moffitt Cancer Center Tampa Florida USA; ^9^ Division of Medical Oncology, Department of Medicine University of Colorado Aurora Colorado USA; ^10^ Division of Medical Oncology, Department of Medicine Roswell Park Comprehensive Cancer Center Buffalo New York USA; ^11^ Department of General Surgery Walter Reed National Military Medical Center Bethesda Maryland USA; ^12^ Division of Hematology/Oncology, Department of Medicine University of Virginia Comprehensive Cancer Center Charlottesville Virginia USA; ^13^ Department of Translational Genomics University of Southern California Los Angeles California USA; ^14^ Division of Hematology/Oncology, Department of Medicine Indiana University Indianapolis Indiana USA; ^15^ Division of Surgical Oncology and Endocrine Surgery, Department of Surgery University of Iowa Iowa City Iowa USA

**Keywords:** biomarkers, colorectal cancer, gene expression regulation, survival

## Abstract

**Background:**

Mutational landscape is prognostic in colorectal cancer (CRC). *Rat sarcoma* (*RAS*) oncogenes, such as *KRAS* and *NRAS*, with driver mutations, portend poor survival outcomes, whereas pathologic mutations in *HRAS* are extremely rare, and their prognostic value remains uncertain.

**Methods:**

This retrospective study analyzed the Oncology Research Information Exchange Network (ORIEN) alliance tumor RNA‐Seq data in Stages II and III CRC to investigate the association between *RAS* gene expression and survival outcomes.

**Results:**

High transcript levels of *HRAS* were associated with superior overall survival (OS). The high *HRAS*‐associated OS benefit was most pronounced in patients with right‐sided primary expressing low *KRAS* transcript levels in the absence of pathologic *KRAS* mutations.

**Conclusions:**

Contrary to the notion that *RAS* family genes are proto‐oncogenic, this study demonstrates that high *HRAS* transcript levels are associated with superior OS in Stages II and III CRC. The potential of *HRAS* as a prognostic biomarker should be explored further.

## 1 Background

Prognostication of early stage colorectal cancer (CRC) at initial diagnosis has traditionally relied on gross tumor anatomy and histology [1]. Due to the high risk of recurrence, the current standard of care treatment for Stage III CRC after surgical resection is adjuvant chemotherapy, whereas for Stage II CRC, due to the overall intermediate risk of recurrence, the decision for adjuvant chemotherapy is individualized based on clinicopathological risk factors such as the anatomy of the primary lesion or the presence of high‐risk histological features [2]. Only a handful of prognostic molecular biomarkers have been reliably utilized in clinical settings, such as *CDX2*, of which the loss of expression leads to inferior disease‐specific survival in Stages II and III colon cancer [3].

The mammalian *rat sarcoma* (*RAS*) gene family or paralogues, *HRAS*, *KRAS*, and *NRAS*, encode GTP‐binding hydrolases that act as a molecular switch of cellular signaling pathways. RAS proteins exist in either an “inactive” conformation when bound to GDP or “active” conformation when mitogenic signals catalyze exchange of the GDP to GTP, which activates a downstream signaling pathway and promotes cell growth. When the bound GTP is hydrolyzed to GDP, the RAS protein converts to its “inactive” state. Oncogenic *RAS* gene mutations result in increased affinity to GTP or loss of GTP hydrolysis [4]. Pathologic *KRAS* mutation is found in approximately 40% of CRC patients, of which approximately 85% occur in codons 12, 13, and 61 [5] and is associated with worse overall survival (OS). The mutation also demonstrates inferior response to standard of care first‐line 5‐fluorouracil (5‐FU)‐based treatments, as well as anti‐EGFR therapy, compared to those harboring wild‐type *KRAS* [6]. *NRAS* mutation is present in approximately 5% of colon cancers and is associated with poor survival outcomes. *HRAS* mutation is extremely rare in CRC, and its prognostic implication remains unclear to date [7, 8]. The prognostic implication of *RAS* paralogue gene activity at the transcript level is also obscure.

Interestingly, there is increasing evidence suggesting that different oncogenic *RAS* paralogues may associate with distinct biological phenotypes [9]. In a mouse model study, *KRAS* ^G12D^ promoted hyperproliferation, whereas *NRAS* ^G12D^ did not affect proliferation but instead suppressed apoptosis of colonic epithelium [10]. A comprehensive review of mutation profiles in advanced CRC patients demonstrated a significantly higher frequency of *HRAS* mutations in the high tumor mutation burden (TMB) tumors compared to low TMB tumors [11]. Furthermore, experimental evidence suggests that proto‐oncogene (or wild‐type) *RAS* signaling in cancer cells harboring an oncogenic *RAS* mutation also contributes to cellular proliferation that is independent from the coexisting oncogenic *RAS* mutation [12], which underscores the potential therapeutic implications of *RAS* proto‐oncogenes.

The aim of this study was to investigate the prognostic implications of *HRAS* in Stages II and III CRC and retrospectively analyzed the transcriptional expression profiles of the RAS family using the tissue RNA‐Seq database of CRC patients. The Cancer Genome Atlas (TCGA) is considered the largest and most comprehensive cancer genomic dataset available. Among the TCGA colorectal adenocarcinoma (COADREAD) cohort, 372 Stages II and III CRC cases with primary tumor RNA‐Seq and censorship data were accessible, which was deemed sub‐optimally small for the purpose of this study. In addition, the original publication of the TCGA‐COADREAD project dates back to 2012 [13] and may not reflect recent survival trends among CRC patients. Therefore, the clinical and RNA‐Seq data from Stages II and III CRC patients enrolled in the Oncology Research Information Exchange Network (ORIEN) AVATAR program was selected as the primary dataset for this study.

## 2 Methods

ORIEN is an ongoing multicenter collaboration between 19 cancer centers in the United States, in which cancer patients are prospectively enrolled in the Total Cancer Care protocol, approved by individual institutions' review board, launched in 2016. Patients with available tumor tissues for molecular profiling are included in the ORIEN AVATAR program. Clinicopathological, treatment, and outcome data are abstracted and periodically updated by individual sites. All molecular profiling and data management are maintained by Aster Insights in collaboration with all participating cancer centers. All clinical and molecular data are de‐identified and made available to investigators from participating institutions upon request and study approval.

From the ORIEN AVATAR cohort, 734 patients with Stages II and III CRC were identified as of April 16, 2024 (Table S1). Primary (*n* = 560) or metastatic (*n* = 174, if primary tumor specimen is not available) tumor tissue whole‐exome sequencing and RNA‐Seq data and their associated clinical data were retrieved. Combat‐Seq from the sva R package was used for transcript count batch correction [14]. Samples that belonged to a batch group with a single sample were excluded. Batch‐corrected transcript counts were normalized to transcripts per million (TPM).

Clinicopathological and batch‐corrected primary tumor tissue RNA‐Seq data (V2 RSEM) of 390 Stages II and III CRC patients enrolled in TCGA‐COADREAD Pan Cancer project were retrieved from the cBio cancer genomics portal [15]. Censorship information was available for 372 cases.

Log‐rank *p*‐value and hazard ratio (HR) were derived from Kaplan–Meier survival analysis. All statistical analyses were performed using GraphPad Prism software.

## 3 Results

**FIGURE 1:**
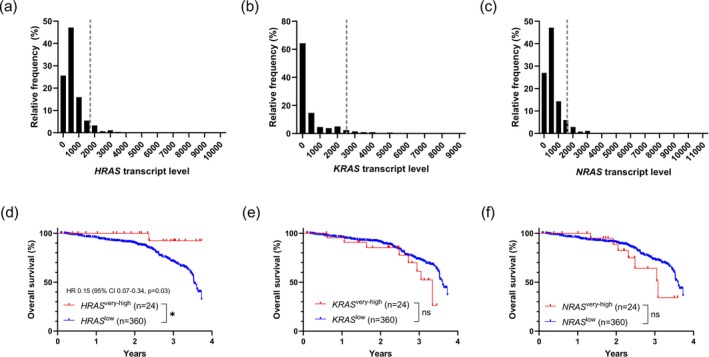
Distribution of (a) *HRAS*, (b) *KRAS*, and (c) *NRAS* transcript levels in combined Stages II and III CRC. Gray vertical dashed lines represent the “very high” or the “top 5%” transcript level cutoffs. Corresponding Kaplan–Meier OS analysis at 3.7 years by (d) *HRAS*, (e) *KRAS*, and (f) *NRAS* transcript levels. ns, Not statistically significant.

Transcript levels of *HRAS*, *KRAS*, and *NRAS* in Stages II and III CRC were unimodally distributed (Figure 1a–c). The 3.7‐year HR for death in patients with “very high” or the “top 5%” *HRAS* transcript level (*n* = 24) compared to the rest (*n* = 360) was 0.15 (95% CI: 0.07–0.34, *p* = 0.03; Figure 1d). However, no significant differential HR was seen in patients with the top 5% *KRAS* (Figure 1e) or *NRAS* (Figure 1f) transcript levels. When patients with primary tumor RNA‐Seq data were analyzed exclusively, a similar trend towards improved 3.7‐year OS associated with “very high” *HRAS* transcript levels was observed, although it did not reach statistical significance (Figure S1a,d). Again, no significant differential HR was associated with “very high” *KRAS* or *NRAS* (Figure S1b,c,e,f).

To identify clinicopathological factors which may contribute to the superior OS observed in patients with “very high” *HRAS* transcript levels, Cox regression analysis was performed, which further revealed Stage II versus III with HR 0.47 (95% CI: 0.28–0.75, *p* = 0.002) and male versus female sex with HR 1.60 (95% CI: 1.04–2.48, *p* = 0.033) as significant prognostic parameters in this patient population (Table 1). “Very high” *HRAS* transcript levels trended towards better survival, although they did not reach statistical significance (HR 0.14, 95% CI: 0.01–0.63, *p* = 0.051). The “very high” *HRAS* transcript level‐associated superior OS was preserved within Stages II and III (Figure S2a,b) as well as within male and female sex (Figure S2c,d), respectively, although statistical significance was not reached, likely due to insufficient sample size. Notably, prior exposure to perioperative 5‐FU‐based chemotherapy was not identified as a significant parameter.

**TABLE 1:** Cox regression analysis on overall survival of Stages II and III colorectal cancer at 3.7 years.

Variable	HR	95% CI	*p* value
Gender (male vs. female)	1.599	1.043 to 2.478	0.033*
Perioperative 5‐FU	1.083	0.7028 to 1.663	0.7154
*KRAS*(+) or *BRAF*(+) or *NRAS*(+)^a^	1.247	0.8153 to 1.911	0.3089
Pathological TNM stage (Stage II vs. III)	0.467	0.2820 to 0.7470	0.0021*
Tumor sidedness (left vs. right)	1.13	0.7289 to 1.758	0.5862
Tumor sidedness (not specified vs. right)	1.743	0.5173 to 4.410	0.2968
*HRAS* transcript level (very high vs. not very high)	0.1389	0.007847 to 0.6337	0.0508

*Note:* *p < 0.05.

^a^ *KRAS* pathologic mutation (codon 12, 13, 61) or *BRAF* mutation (any) or *NRAS* mutation (any exon mutation).

Interactions between the *RAS* family genes have been hypothesized and reported previously in an animal cancer model study [16]. To investigate whether *KRAS* transcript levels had any influence on the *HRAS* expression‐associated differential OS, Stages II and III CRC patients were dichotomized by *KRAS* transcript levels. To avoid confounding effects of underlying *RAS* pathway gene mutation on survival outcomes, patients with pathologic mutations in *KRAS* (codons 12, 13, and 61), *NRAS* (any exonic), or *BRAF* (any) were excluded. Of note, *BRAF* encodes for serine/threonine kinase that acts downstream of *RAS* paralogues, and its oncogenic mutations are prevalent in approximately 10% of CRC [17]. To optimize statistical power, the cohort median transcript level of *HRAS* and *KRAS* was used as a cutoff to define “high” versus “low” *HRAS* and *KRAS* transcript levels, respectively (Figure S3a). The 5‐year HR for death in patients with “high” *HRAS* transcript levels with concomitant “low” *KRAS* transcript levels (*n* = 77) compared to those with “high” *KRAS* transcript levels (*n* = 53) was 0.42 (95% CI: 0.18–0.96, *p* = 0.03; Figure S3b). On the other hand, “low” and “high” *KRAS* transcript levels did not differentiate HR for death in patients with “low” *HRAS* transcript levels (Figure S3c). These findings remained consistent even when the analysis was limited to patients with primary tumor RNA‐Seq data. The 5‐year HR for death in patients with “high” *HRAS* transcript levels with concomitant “low” *KRAS* transcript levels (*n* = 62) compared to those with “high” *KRAS* transcript levels (*n* = 48) was 0.22 (95% CI: 0.08–0.62, *p* = 0.004; Figure S4a,b). However, “low” and “high” *KRAS* transcript levels did not differentiate HR for death in patients with “low” *HRAS* transcript levels (Figure S4c).

When analyses were performed in patients harboring pathologic *KRAS* mutations (codons 12, 13, and 61) but not *NRAS* or *BRAF* mutations, “low” and “high” *KRAS* transcript levels no longer differentiated the 5‐year HR for death in patients with “low” and “high” *HRAS* transcript levels (Figure S5a–c). Again, these findings remained consistent even when the analysis was limited to patients with primary tumor RNA‐Seq data (Figure S6a–c).

Similar analyses were performed to investigate whether *NRAS* transcript levels had any influence on the *HRAS* expression‐associated differential OS, but “low” and “high” *NRAS* transcript levels did not differentiate the 5‐year HR for death in patients with “low” and “high” *HRAS* transcript levels (Figure S7a–c). Again, these findings remained consistent even when the analysis was limited to patients with primary tumor RNA‐Seq data (Figure S8a–c).

To further identify clinicopathological factors which may contribute to the superior 5‐year OS observed in Stages II and III CRC patients with “high” *HRAS* transcript levels with no pathologic *KRAS*, *NRAS*, or *BRAF* mutations, Cox regression analysis was performed, which revealed left versus right primary tumor sidedness with HR for death at 3.1 (95% CI: 1.13–10.01, *p* = 0.039) as a significant prognostic parameter in this subpopulation (Table S2). Subsequent Kaplan–Meier survival analysis confirmed that patients expressing “high” *HRAS* with concomitant “low” *KRAS* transcript levels were associated with superior OS compared to those with concomitant “high” *KRAS* transcript levels with HR for death at 0.17 (95% CI: 0.03–0.99, *p* = 0.06) when patients had right‐sided primary tumor (Figure S9a), but not when patients had left‐sided primary tumor (Figure S9b). When patients with primary tumor RNA‐Seq data were analyzed exclusively, a similar trend towards improved 5‐year OS associated with “high” *HRAS* with concomitant “low” *KRAS* was present that is more pronounced in patients with right‐sided primary tumor (Figure S10a), compared to those with left‐sided primary tumor (Figure S10b), although statistical significance was not reached.

To validate these findings in an independent dataset, the association between *RAS* transcript levels and OS was investigated in Stages II and III CRC patients with no pathologic *KRAS*, *NRAS*, or *BRAF* mutation in the TCGA‐COADREAD cohort, although analyses were limited due to small sample size. All RNA‐Seq tissue specimens were from the primary tumor. Again, transcript levels of *HRAS*, *KRAS*, and *NRAS* were unimodally distributed (Figure S11a–c). The 40‐month HR for death in patients with “very high” (defined as the “top 15%” due to smaller sample size) *HRAS* transcript level (*n* = 22) was not significantly different compared to the rest (*n* = 140), although there was a trend towards improved OS (Figure S11d), which is consistent with the observation from the ORIEN dataset. Similarly, no significant differential HR was seen in patients with the top 15% *KRAS* (Figure S11e) or *NRAS* transcript levels (Figure S11f). Next, the influence of *KRAS* transcript level on *HRAS*‐dependent OS was analyzed. Given skewness of the transcript level distributions, the cutoff to define “high” versus “low” *KRAS* transcript levels was set differently for “high” and “low” *HRAS* level groups (Figure S12a). The 40‐month HR for death in patients with “high” *HRAS* transcript levels and concomitant “low” *KRAS* transcript levels (*n* = 7) was not significantly different compared to those with “high” *KRAS* transcript levels (*n* = 68), but again there was a trend towards improved OS (Figure S12b). On the other hand, “low” and “high” *KRAS* transcript levels did not differentiate the HR for death in patients with “low” *HRAS* transcript levels (Figure S12c).

## 4 Discussion

Strikingly, in Stages II and III CRC, superior OS was associated with the subgroup of patients exhibiting high *HRAS* transcript levels compared to those expressing low *HRAS* transcript levels, which was most pronounced in patients with concomitant low *KRAS* transcript levels without pathologic *KRAS* mutation, with right‐sided primary tumor location. Approximately 24% of the RNA‐Seq data in the ORIEN dataset were from non‐primary tumor specimens, but these findings were consistently present even when primary tumor RNA‐Seq data was analyzed exclusively.

Contrary to the notion that *RAS* family genes are proto‐oncogenic, this study demonstrates that “high” *HRAS* transcript levels are associated with superior OS. The “high” *HRAS*‐associated OS benefit was most pronounced in patients with right‐sided primary tumors concomitantly expressing “low” *KRAS* transcript levels in the absence of pathologic *KRAS* mutations. When expressed at high levels, *HRAS* may counteract the proto‐oncogene *KRAS* but not the oncogene *KRAS*. Antagonistic interaction between the *RAS* paralogues has been previously reported in a KRAS‐mutated lung cancer mouse model study using the CRISPR/Cas9 gene editing system, which demonstrated that HRAS and NRAS suppress tumor proliferation by directly interacting with oncogenic KRAS at the protein level [16]. Our study suggests that the antagonistic relationship between *RAS* paralogue genes may be more prevalent than previously assumed.

There are limitations to this study. First, even though ORIEN is a multicentric cohort, the findings of this study are based on a single dataset. It is reassuring that the survival analyses using a much smaller available dataset (TCGA‐COADREAD) demonstrated a similar trend towards improved survival outcome in the patient group with “high” *HRAS* and “low” *KRAS*, although statistical significance was not reached. Second, this was a retrospective study. Further validation with an independent cohort dataset and prospective investigation is warranted.

Only a handful of gene expression level‐based prognostic biomarkers for CRC are reliably used in clinical settings. Loss of expression of mismatch repair (MMR) genes, namely *MLH1*, *MSH2*, *MSH6*, and *PMS2*, is associated with an excellent response to checkpoint inhibitor immunotherapies. Loss of expression of *CDX2*, which is a caudal‐type homeobox transcription factor, portends a higher risk of recurrence, which warrants adjuvant chemotherapy in Stage II CRC, especially when other recurrence risk factors are absent [3]. Overexpression of *HRAS* was previously associated with responsiveness to lenvatinib in human gastro‐entero‐pancreatic neuroendocrine tumor cell lines [18], but the prognostic implication of *HRAS* expression has not been reported in CRC to date. The potential of *HRAS* as a prognostic biomarker in Stages II and III CRC should be explored further.

## Author Contributions

**Donghyun Kim:** conceptualization, writing – original draft, investigation, formal analysis, writing – review and editing, methodology, visualization. **Saima Sharif:** writing – review and editing, supervision. **Juan Antonio Raygoza Garay:** writing – review and editing, data curation. **Avanish S. Bhakta:** writing – review and editing, resources. **Patrick M. Boland:** writing – review and editing, resources. **Michael J. Cavnar:** writing – review and editing, resources. **Michelle L. Churchman:** writing – review and editing, project administration. **Hassan Hatoum:** writing – review and editing, resources. **Lyen C. Huang:** writing – review and editing, resources. **Joseph Kim:** writing – review and editing, resources. **Richard Kim:** writing – review and editing, resources. **Robert W. Lentz:** writing – review and editing, resources. **Sarbajit Mukherjee:** writing – review and editing, resources. **Mary T. O'Donnell:** writing – review and editing, resources. **Benjamin Quartey:** writing – review and editing, resources. **Matthew J. Reilley:** writing – review and editing, resources. **Robert J. Rounbehler:** writing – review and editing, project administration. **Bodour Salhia:** writing – review and editing, resources. **Bryan P. Schneider:** writing – review and editing, resources. **Carlos H. Chan:** writing – review and editing, supervision, formal analysis, resources, methodology, writing – original draft, conceptualization.

## Ethics Statement

The authors have nothing to report.

## Conflicts of Interest

The authors declare no conflicts of interest.

## Supporting information


**Figure S1:** Distribution of (a) *HRAS*, (b) *KRAS*, and (c) *NRAS* transcript levels in combined Stages II and III CRC patients, *in primary tumor only*. On Gray vertical dashed lines represent the “very high” or the “top 5%” transcript level cutoffs. Corresponding Kaplan–Meier OS analysis at 3.7 years by (d) *HRAS*, (e) *KRAS*, and (f) *NRAS* transcript levels. ns, not statistically significant.

**Figure S2:** Kaplan–Meier OS analysis at 3.7 years by *HRAS* transcript levels in (a) Stage II, (b) Stage III, (c) male‐only in Stages II and III combined, and (d) female‐only in Stages II and III combined CRC patient subgroups. ns, not statistically significant.

**Figure S3:** Kaplan–Meier OS analysis at 5 years by *HRAS* and *KRAS* transcript levels in Stages II and III CRC patients with no pathologic *KRAS*, *NRAS*, or *BRAF* mutations. (a) Scatter plot of *HRAS* and *KRAS* transcript levels; gray dashed lines represent median value, (b) Kaplan–Meier OS analysis in patients with “high” *HRAS* transcript levels by “low” versus “high” *KRAS* transcript levels, and (c) Kaplan–Meier OS analysis in patients with “low” *HRAS* transcript levels by “low” versus “high” *KRAS* transcript levels. ns, not statistically significant.

**Figure S4:** Kaplan–Meier OS analysis at 5 years by *HRAS* and *KRAS* transcript levels in Stages II and III CRC patients with no pathologic *KRAS*, *NRAS*, or *BRAF* mutations, *in primary tumor only*. (a) Scatter plot of *HRAS* and *KRAS* transcript levels; gray dashed lines represent median value, (b) Kaplan–Meier OS analysis in patients with “high” *HRAS* transcript levels by “low” versus “high” *KRAS* transcript levels, and (c) Kaplan–Meier OS analysis in patients with “low” *HRAS* transcript levels by “low” versus “high” *KRAS* transcript levels. ns, not statistically significant.

**Figure S5:** Kaplan–Meier OS analysis at 5 years by *HRAS* and *KRAS* transcript levels in Stages II and III CRC patients with pathologic KRAS mutation but without *NRAS* or *BRAF* mutations. (a) Scatter plot of *HRAS* and *KRAS* transcript levels; gray dashed lines represent median value, (b) Kaplan–Meier OS analysis in patients with “high” *HRAS* transcript levels by “low” versus “high” *KRAS* transcript levels, and (c) Kaplan–Meier OS analysis in patients with “low” *HRAS* transcript levels by “low” versus “high” *KRAS* transcript levels. ns, not statistically significant.

**Figure S6:** Kaplan–Meier OS analysis at 5 years by *HRAS* and *KRAS* transcript levels in Stages II and III CRC patients with pathologic KRAS mutation but without *NRAS* or *BRAF* mutations, *in primary tumor only*. (a) Scatter plot of *HRAS* and *KRAS* transcript levels; gray dashed lines represent median value, (b) Kaplan–Meier OS analysis in patients with “high” *HRAS* transcript levels by “low” versus “high” *KRAS* transcript levels, and (c) Kaplan–Meier OS analysis in patients with “low” *HRAS* transcript levels by “low” versus “high” *KRAS* transcript levels. ns, not statistically significant.

**Figure S7:** Kaplan–Meier OS analysis at 5 years by *HRAS* and *NRAS* transcript levels in Stages II and III CRC patients with no pathologic *KRAS*, *NRAS*, or *BRAF* mutations. (a) Scatter plot of *HRAS* and *NRAS* transcript levels; gray dashed lines represent median value, (b) Kaplan–Meier OS analysis in patients with “high” *HRAS* transcript levels by “low” versus “high” *NRAS* transcript levels, and (c) Kaplan–Meier OS analysis in patients with “low” *HRAS* transcript levels by “low” versus “high” *NRAS* transcript levels. ns, not statistically significant.

**Figure S8:** Kaplan–Meier OS analysis at 5 years by *HRAS* and *NRAS* transcript levels in Stages II and III CRC patients with no pathologic *KRAS*, *NRAS*, or *BRAF* mutations, *in primary tumor only*. (a) Scatter plot of *HRAS* and *NRAS* transcript levels; gray dashed lines represent median value, (b) Kaplan–Meier OS analysis in patients with “high” *HRAS* transcript levels by “low” versus “high” *NRAS* transcript levels, and (c) Kaplan–Meier OS analysis in patients with “low” *HRAS* transcript levels by “low” versus “high” *NRAS* transcript levels. ns, not statistically significant.

**Figure S9:** Kaplan–Meier OS analysis at 5 years in patients with high *HRAS* transcript levels by “low” versus “high” *KRAS* transcript levels with (a) right‐sided primary tumor and (b) left‐sided primary tumor in the absence of pathologic *KRAS*, *NRAS* and *BRAF* mutations. ns, not statistically significant.

**Figure S10:** Kaplan–Meier OS analysis at 5 years in patients with high *HRAS* transcript levels by “low” versus “high” *KRAS* transcript levels with (a) right‐sided primary tumor and (b) left‐sided primary tumor in the absence of pathologic *KRAS*, *NRAS* and *BRAF* mutations, *in primary tumor only*. ns, not statistically significant.

**Figure S11:** Distribution of (a) *HRAS*, (b) *KRAS*, and (c) *NRAS* transcript levels in combined Stages II and III CRC of TCGA‐COADREAD cohort. Gray vertical dashed lines represent the “very high” or the “top 15%” transcript level cutoffs. Corresponding Kaplan–Meier OS analysis at 40 months by (d) *HRAS*, (e) *KRAS*, and (f) *NRAS* transcript levels. ns, not statistically significant.

**Figure S12:** Kaplan–Meier OS analysis at 40 months by *HRAS* and *KRAS* transcript levels in Stages II and III CRC patients of TCGA‐COADREAD cohort with no pathologic *KRAS*, *NRAS*, or *BRAF* mutations. (a) Scatter plot of *HRAS* and *KRAS* transcript levels; gray vertical dashed line represents median value, and gray horizontal dashed lines represent selected cutoffs, (b) Kaplan–Meier OS analysis in patients with “high” *HRAS* transcript levels by “low” versus “high” *KRAS* transcript levels, and (c) Kaplan–Meier OS analysis in patients with “low” *HRAS* transcript levels by “low” versus “high” *KRAS* transcript levels. ns, not statistically significant.

**Table S1:** ORIEN AVATAR Stages II and III CRC patient summary.

**Table S2:** Cox regression analysis on overall survival of Stages II and III colorectal cancer with high *HRAS* transcript expression in *KRAS*(−); *BRAF*(−); *NRAS*(−) genotypic background at 5 years.

## Data Availability

The data that support the findings of this study are available from ORIEN. Restrictions apply to the availability of these data, which were used under license for this study. Data are available from https://www.oriencancer.org/ with the permission of ORIEN.
