# Supplementary figures and images for: Proto‐Oncogene HRAS Transcript Level and Overall Survival in Stages II and III Colorectal Cancer
Source: Cancer Med. 2025 Jul 31;14(15):e71114. doi: 10.1002/cam4.71114 (PMC12311480; doi:10.1002/cam4.71114)

# Supplementary Figure 1

## Primary tumor only

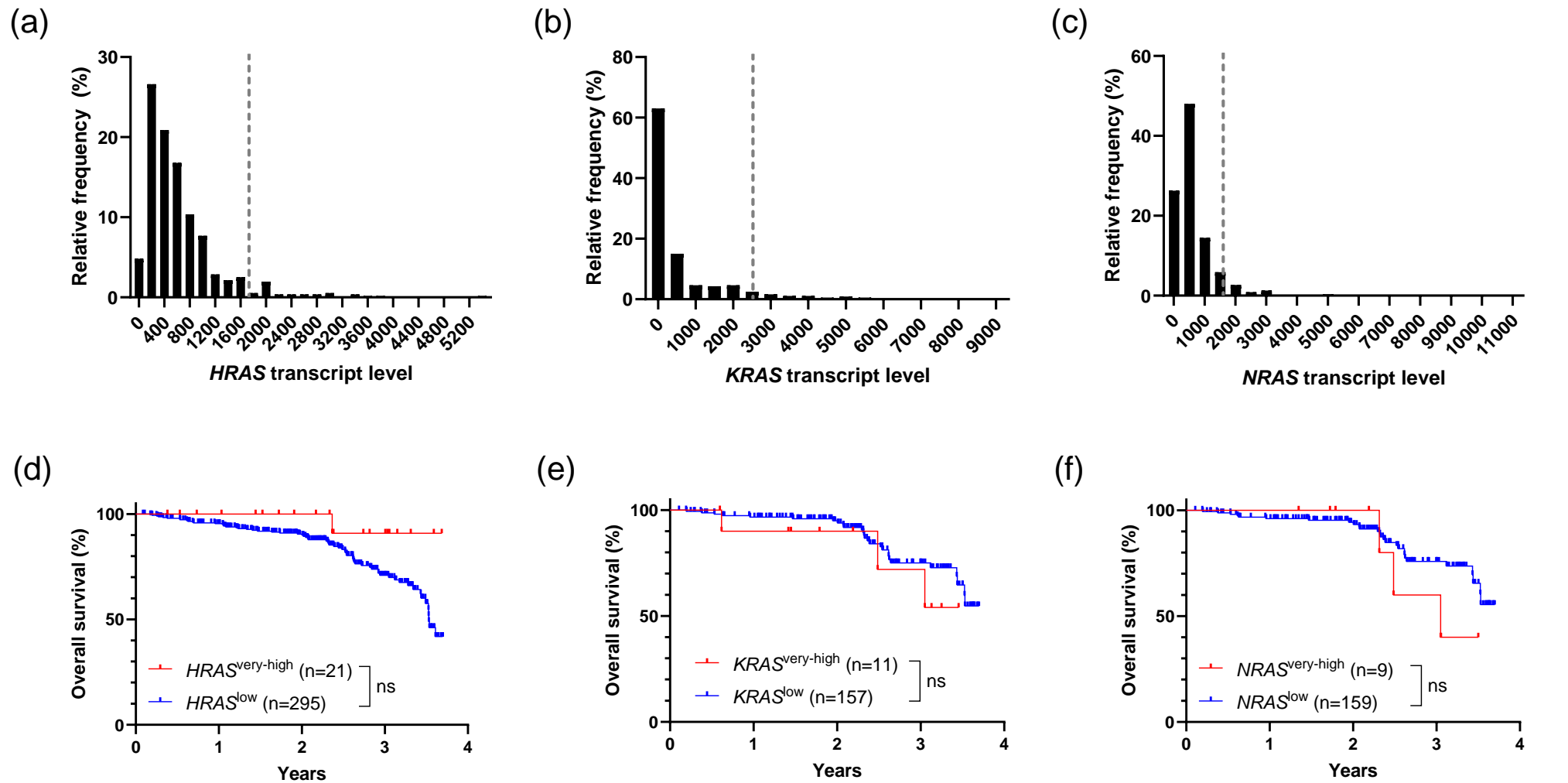

Supplement: Supplementary file 1 — Figure S1: Distribution of (a) HRAS, (b) KRAS, and (c) NRAS transcript levels in combined Stages II and III CRC patients, in primary tumor only. On Gray vertical dashed lines represent the “very high” or the “top 5%” transcript level cutoffs. Corresponding Kaplan–Meier OS analysis at 3.7 years by (d) HRAS, (e) KRAS, and (f) NRAS transcript levels. ns, not statistically significant. [file CAM4-14-e71114-s011.pdf]

# Supplementary Figure 2

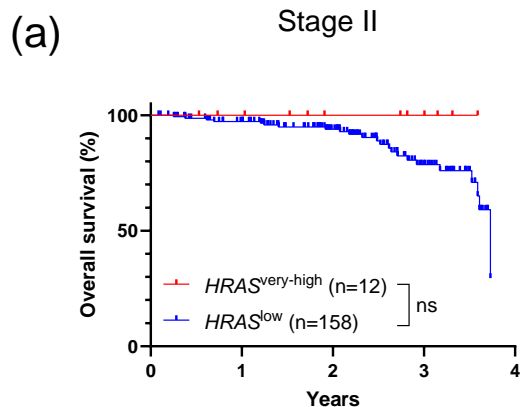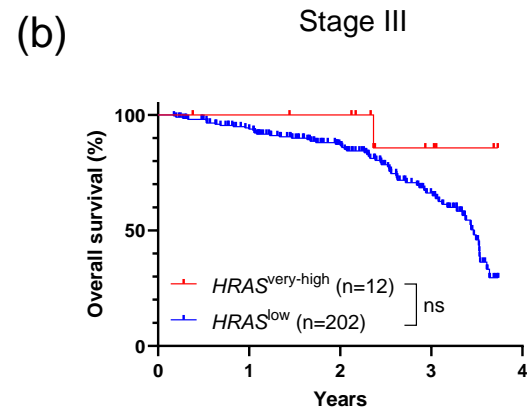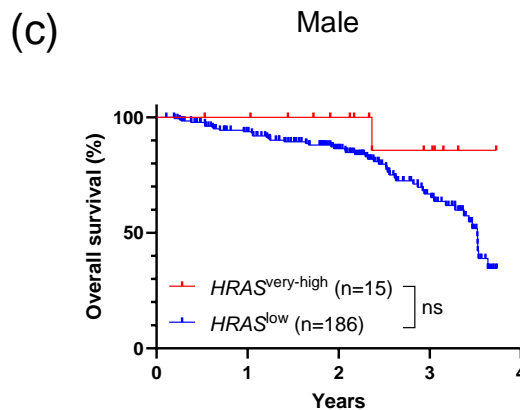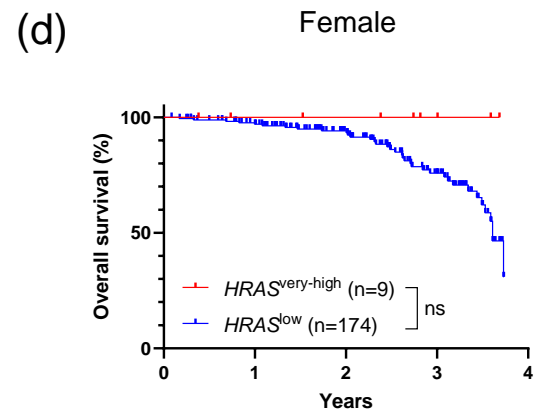

Supplement: Supplementary file 2 — Figure S2: Kaplan–Meier OS analysis at 3.7 years by HRAS transcript levels in (a) Stage II, (b) Stage III, (c) male‐only in Stages II and III combined, and (d) female‐only in Stages II and III combined CRC patient subgroups. ns, not statistically significant. [file CAM4-14-e71114-s014.pdf]

# Supplementary Figure 3

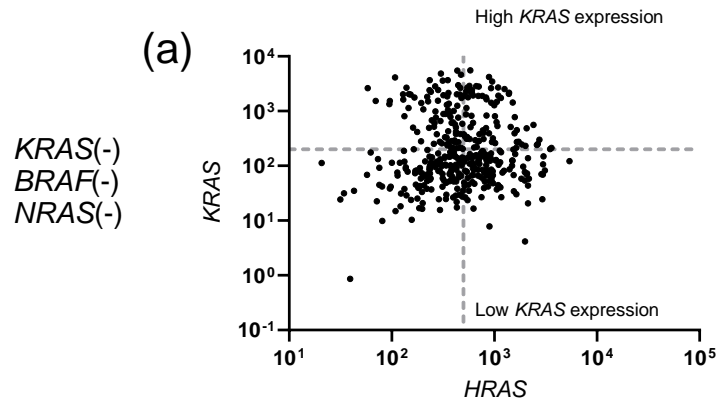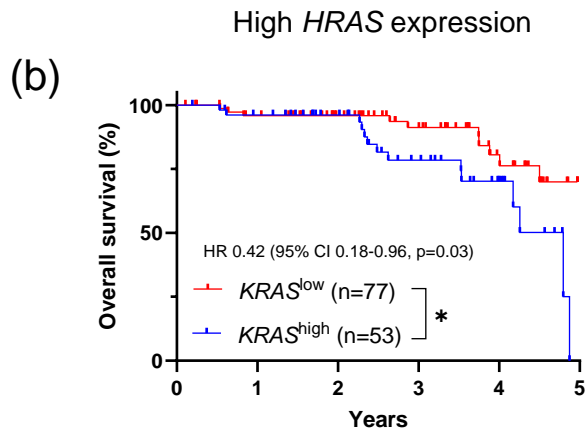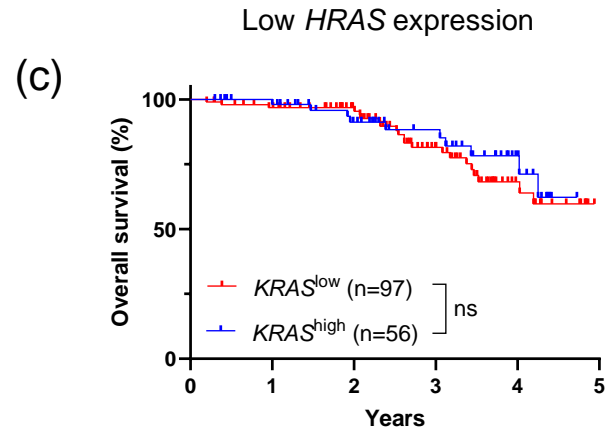

Supplement: Supplementary file 3 — Figure S3: Kaplan–Meier OS analysis at 5 years by HRAS and KRAS transcript levels in Stages II and III CRC patients with no pathologic KRAS, NRAS, or BRAF mutations. (a) Scatter plot of HRAS and KRAS transcript levels; gray dashed lines represent median value, (b) Kaplan–Meier OS analysis in patients with “high” HRAS transcript levels by “low” versus “high” KRAS transcript levels, and (c) Kaplan–Meier OS analysis in patients with “low” HRAS transcript levels by “low” versus “high” KRAS transcript levels. ns, not statistically significant. [file CAM4-14-e71114-s001.pdf]

# Supplementary Figure 4

## Primary tumor only

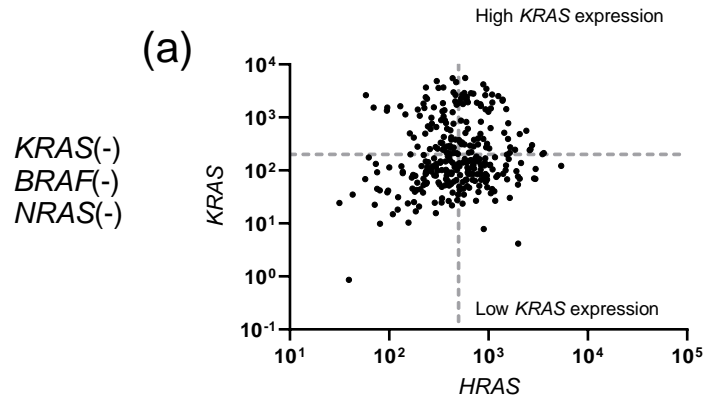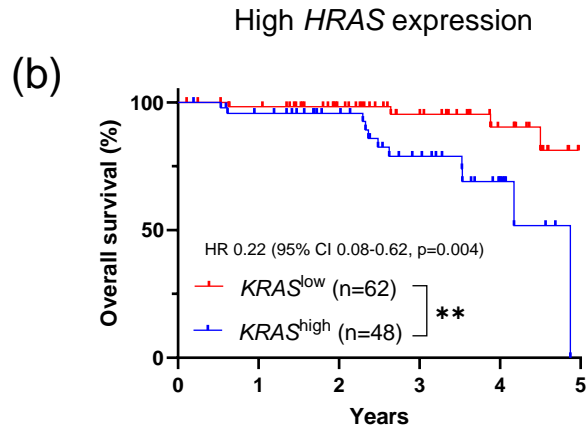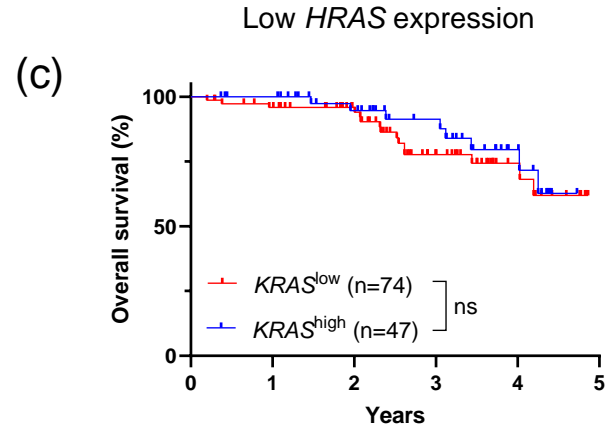

Supplement: Supplementary file 4 — Figure S4: Kaplan–Meier OS analysis at 5 years by HRAS and KRAS transcript levels in Stages II and III CRC patients with no pathologic KRAS, NRAS, or BRAF mutations, in primary tumor only. (a) Scatter plot of HRAS and KRAS transcript levels; gray dashed lines represent median value, (b) Kaplan–Meier OS analysis in patients with “high” HRAS transcript levels by “low” versus “high” KRAS transcript levels, and (c) Kaplan–Meier OS analysis in patients with “low” HRAS transcript levels by “low” versus “high” KRAS transcript levels. ns, not statistically significant. [file CAM4-14-e71114-s007.pdf]

# Supplementary Figure 5

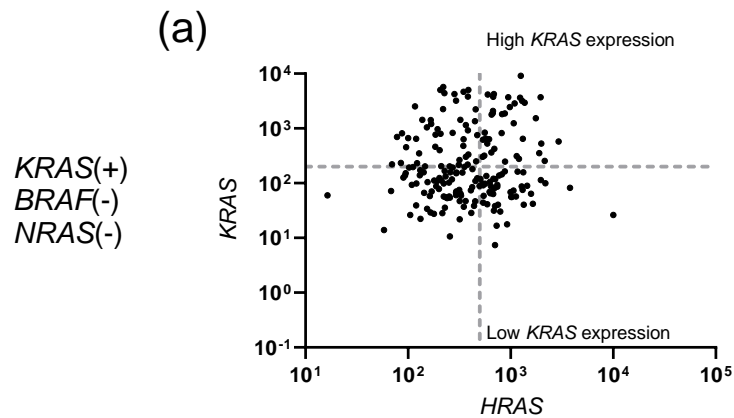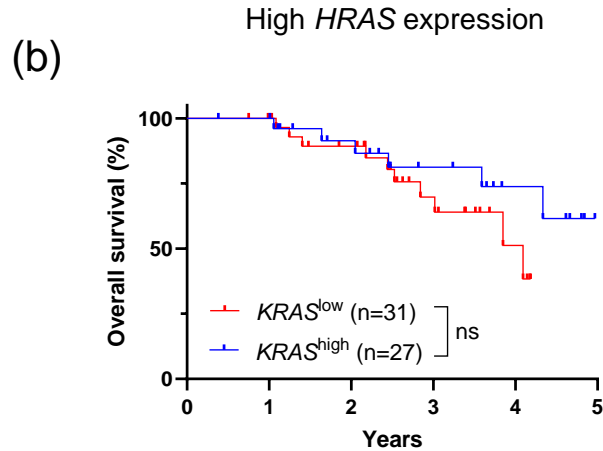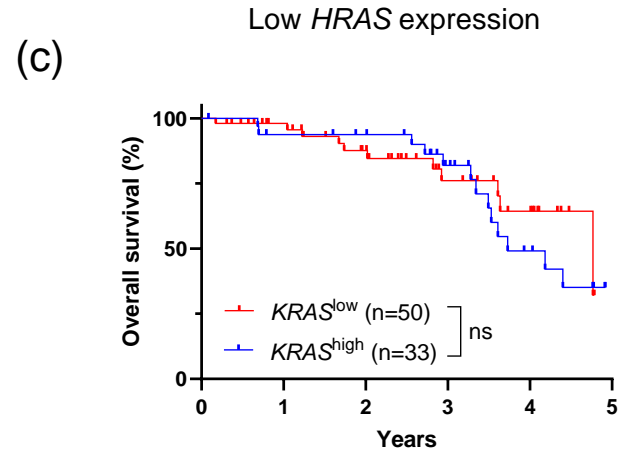

Supplement: Supplementary file 5 — Figure S5: Kaplan–Meier OS analysis at 5 years by HRAS and KRAS transcript levels in Stages II and III CRC patients with pathologic KRAS mutation but without NRAS or BRAF mutations. (a) Scatter plot of HRAS and KRAS transcript levels; gray dashed lines represent median value, (b) Kaplan–Meier OS analysis in patients with “high” HRAS transcript levels by “low” versus “high” KRAS transcript levels, and (c) Kaplan–Meier OS analysis in patients with “low” HRAS transcript levels by “low” versus “high” KRAS transcript levels. ns, not statistically significant. [file CAM4-14-e71114-s002.pdf]

# Supplementary Figure 6

Primary tumor only

(a)

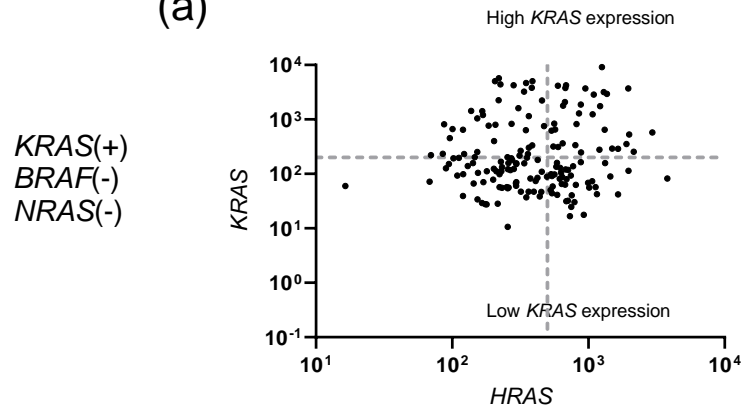

(b)

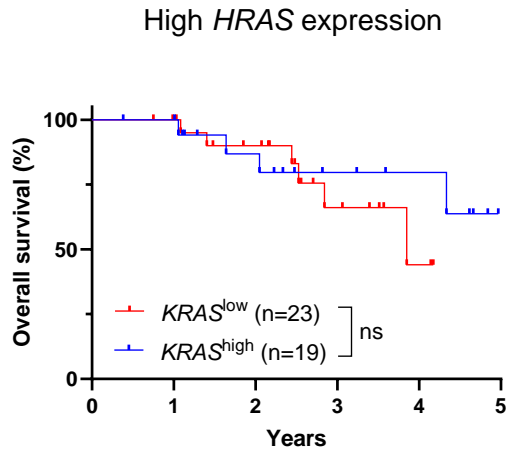

(c)

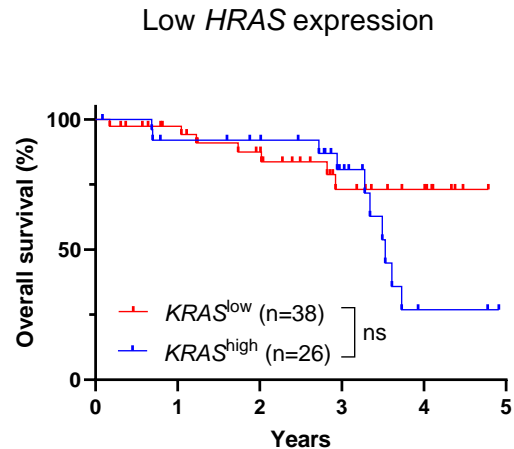

Supplement: Supplementary file 6 — Figure S6: Kaplan–Meier OS analysis at 5 years by HRAS and KRAS transcript levels in Stages II and III CRC patients with pathologic KRAS mutation but without NRAS or BRAF mutations, in primary tumor only. (a) Scatter plot of HRAS and KRAS transcript levels; gray dashed lines represent median value, (b) Kaplan–Meier OS analysis in patients with “high” HRAS transcript levels by “low” versus “high” KRAS transcript levels, and (c) Kaplan–Meier OS analysis in patients with “low” HRAS transcript levels by “low” versus “high” KRAS transcript levels. ns, not statistically significant. [file CAM4-14-e71114-s003.pdf]

# Supplementary Figure 7

(a)

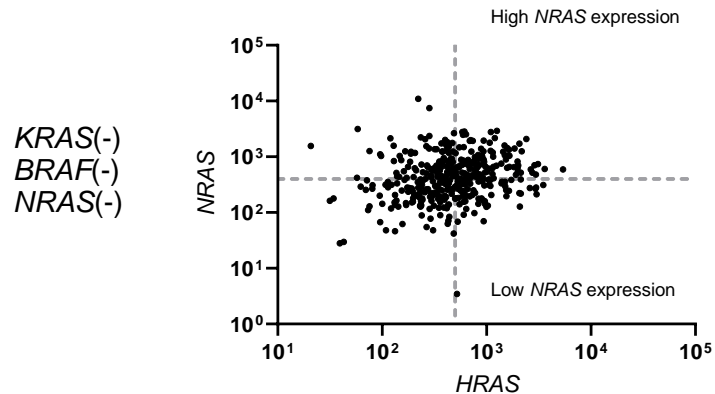

(b)

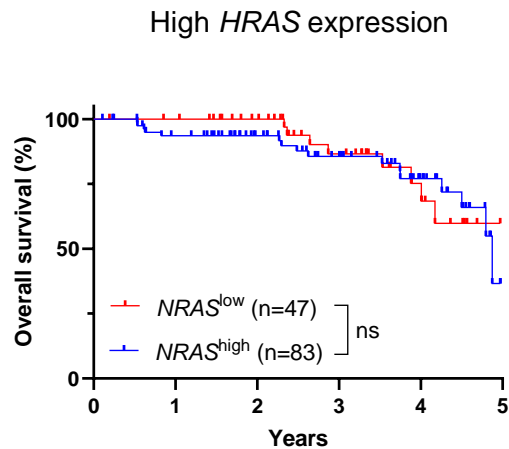

(c)

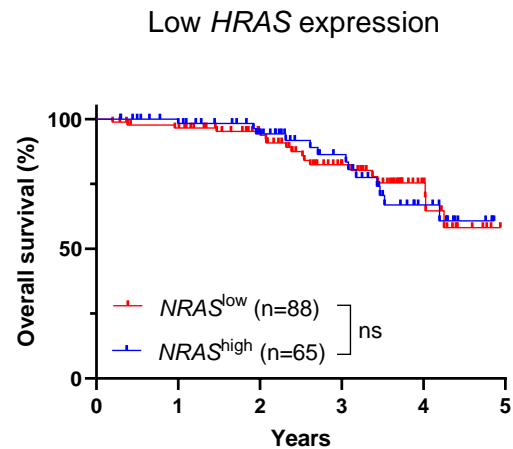

Supplement: Supplementary file 7 — Figure S7: Kaplan–Meier OS analysis at 5 years by HRAS and NRAS transcript levels in Stages II and III CRC patients with no pathologic KRAS, NRAS, or BRAF mutations. (a) Scatter plot of HRAS and NRAS transcript levels; gray dashed lines represent median value, (b) Kaplan–Meier OS analysis in patients with “high” HRAS transcript levels by “low” versus “high” NRAS transcript levels, and (c) Kaplan–Meier OS analysis in patients with “low” HRAS transcript levels by “low” versus “high” NRAS transcript levels. ns, not statistically significant. [file CAM4-14-e71114-s009.pdf]

# Supplementary Figure 8

## Primary tumor only

(a)

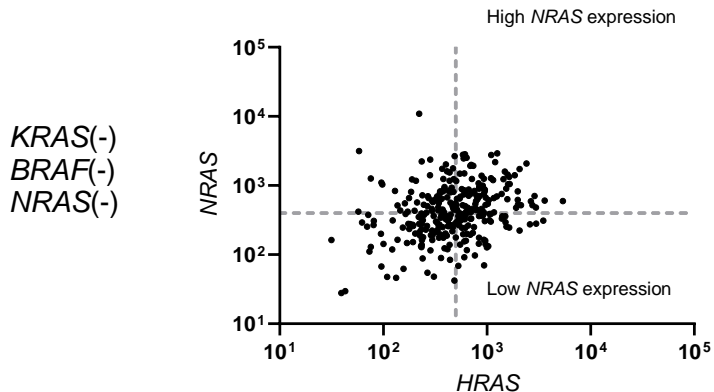

(b)

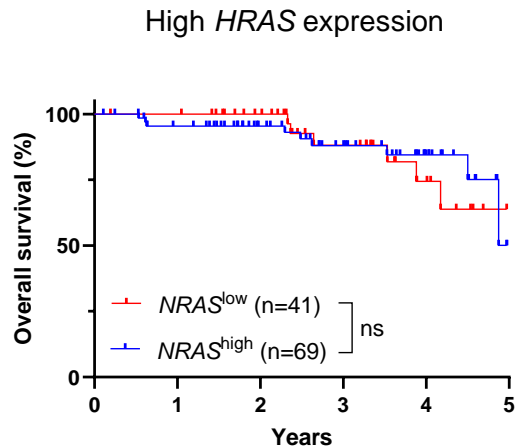

(c)

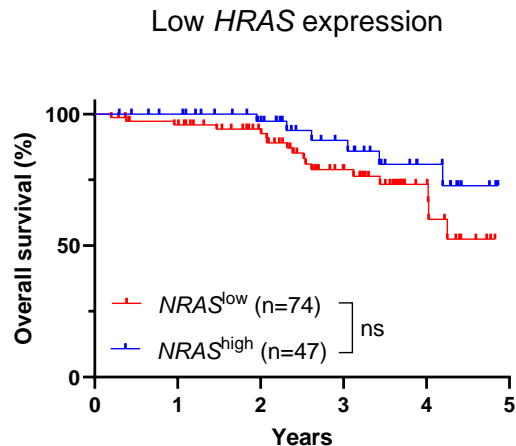

Supplement: Supplementary file 8 — Figure S8: Kaplan–Meier OS analysis at 5 years by HRAS and NRAS transcript levels in Stages II and III CRC patients with no pathologic KRAS, NRAS, or BRAF mutations, in primary tumor only. (a) Scatter plot of HRAS and NRAS transcript levels; gray dashed lines represent median value, (b) Kaplan–Meier OS analysis in patients with “high” HRAS transcript levels by “low” versus “high” NRAS transcript levels, and (c) Kaplan–Meier OS analysis in patients with “low” HRAS transcript levels by “low” versus “high” NRAS transcript levels. ns, not statistically significant. [file CAM4-14-e71114-s005.pdf]

# Supplementary Figure 10

## Primary tumor only

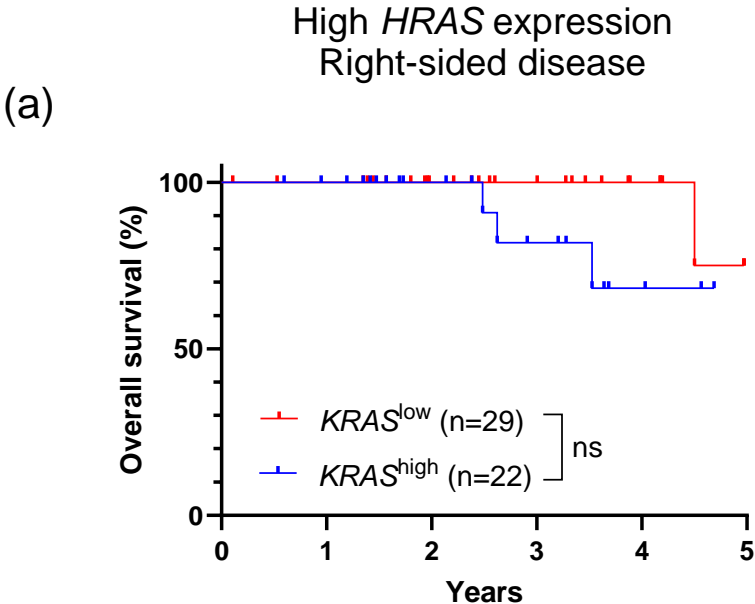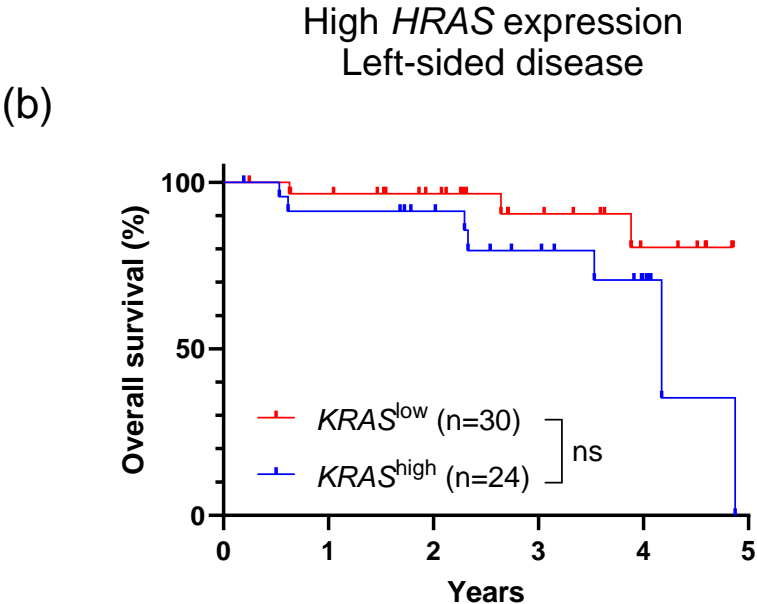

*KRAS*(-)  
*BRAF*(-)  
*NRAS*(-)

Supplement: Supplementary file 10 — Figure S10: Kaplan–Meier OS analysis at 5 years in patients with high HRAS transcript levels by “low” versus “high” KRAS transcript levels with (a) right‐sided primary tumor and (b) left‐sided primary tumor in the absence of pathologic KRAS, NRAS and BRAF mutations, in primary tumor only. ns, not statistically significant. [file CAM4-14-e71114-s004.pdf]

# Supplementary Figure 11

Stage II and III KRAS(-) BRAF(-) NRAS(-) patients in TCGA-COADREAD cohort

(a)

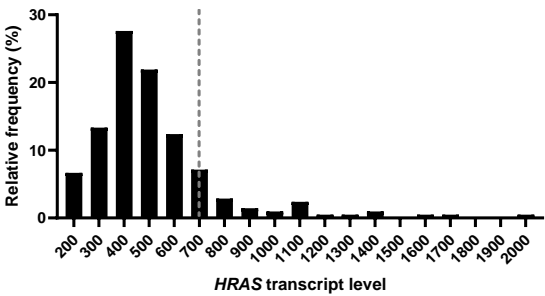

(b)

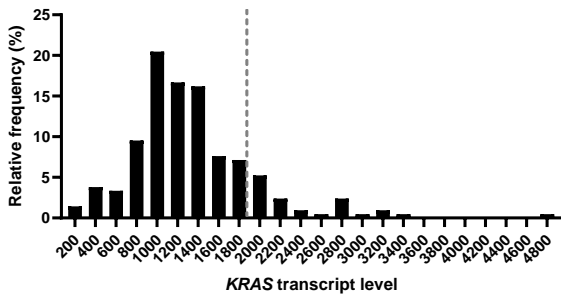

(c)

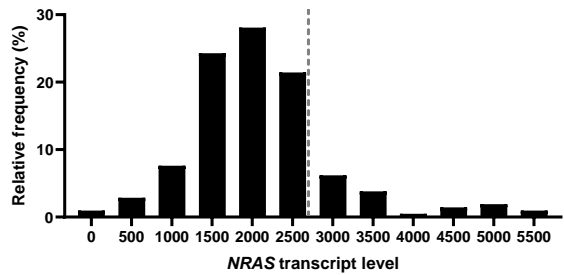

(d)

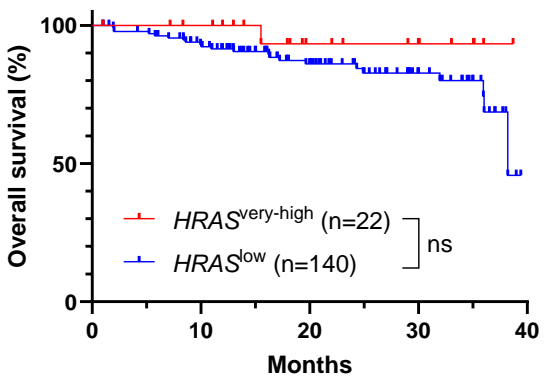

(e)

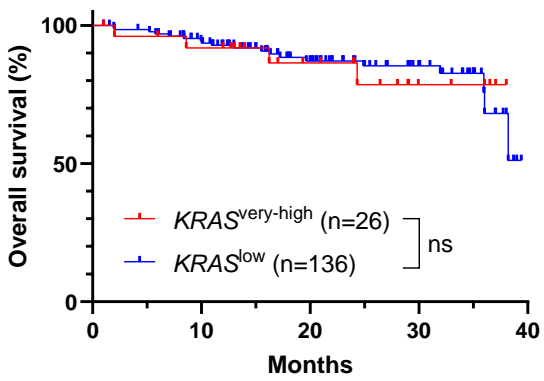

(f)

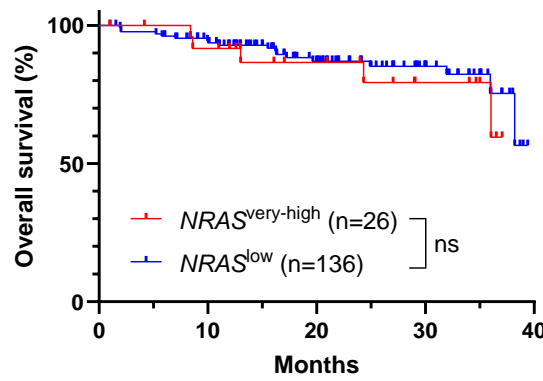

Supplement: Supplementary file 11 — Figure S11: Distribution of (a) HRAS, (b) KRAS, and (c) NRAS transcript levels in combined Stages II and III CRC of TCGA‐COADREAD cohort. Gray vertical dashed lines represent the “very high” or the “top 15%” transcript level cutoffs. Corresponding Kaplan–Meier OS analysis at 40 months by (d) HRAS, (e) KRAS, and (f) NRAS transcript levels. ns, not statistically significant. [file CAM4-14-e71114-s013.pdf]

# Supplementary Figure 12

Stage II and III KRAS(-) BRAF(-) NRAS(-) patients in TCGA-COADREAD cohort

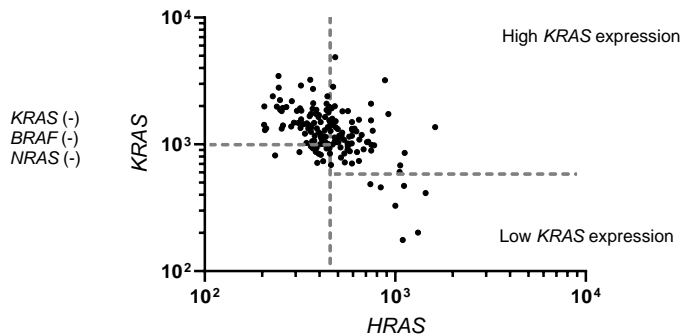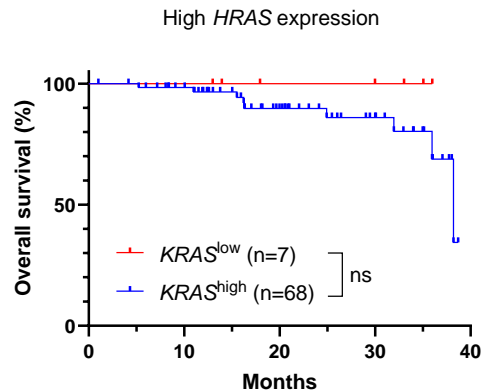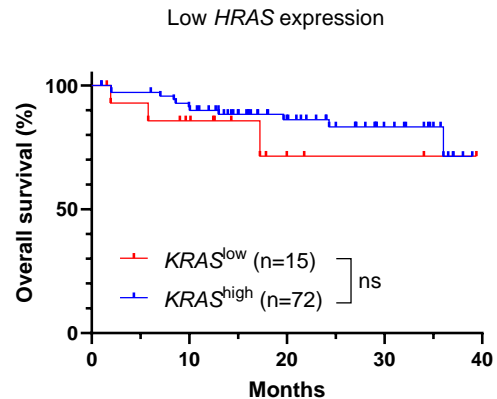

Supplement: Supplementary file 12 — Figure S12: Kaplan–Meier OS analysis at 40 months by HRAS and KRAS transcript levels in Stages II and III CRC patients of TCGA‐COADREAD cohort with no pathologic KRAS, NRAS, or BRAF mutations. (a) Scatter plot of HRAS and KRAS transcript levels; gray vertical dashed line represents median value, and gray horizontal dashed lines represent selected cutoffs, (b) Kaplan–Meier OS analysis in patients with “high” HRAS transcript levels by “low” versus “high” KRAS transcript levels, and (c) Kaplan–Meier OS analysis in patients with “low” HRAS transcript levels by “low” versus “high” KRAS transcript levels. ns, not statistically significant. [file CAM4-14-e71114-s010.pdf]
