# Supplementary material for: Proto‐Oncogene HRAS Transcript Level and Overall Survival in Stages II and III Colorectal Cancer
Source: Cancer Med. 2025 Jul 31;14(15):e71114. doi: 10.1002/cam4.71114 (PMC12311480; doi:10.1002/cam4.71114)

Supplementary Figure 9

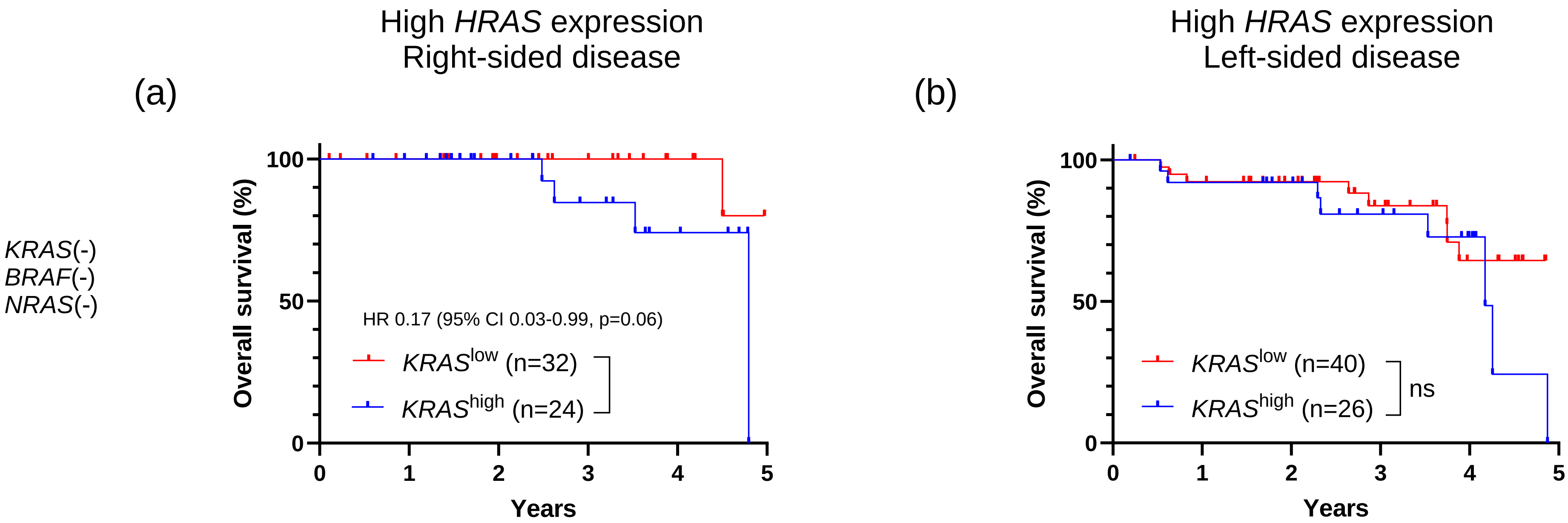

Supplement: Supplementary file 9 — Figure S9: Kaplan–Meier OS analysis at 5 years in patients with high HRAS transcript levels by “low” versus “high” KRAS transcript levels with (a) right‐sided primary tumor and (b) left‐sided primary tumor in the absence of pathologic KRAS, NRAS and BRAF mutations. ns, not statistically significant. [file CAM4-14-e71114-s012.pdf]
