# Supplementary material for: Proto‐Oncogene HRAS Transcript Level and Overall Survival in Stages II and III Colorectal Cancer
Source: Cancer Med. 2025 Jul 31;14(15):e71114. doi: 10.1002/cam4.71114 (PMC12311480; doi:10.1002/cam4.71114)
Supplement: Supplementary file 13 — Table S1: ORIEN AVATAR Stages II and III CRC patient summary. [file CAM4-14-e71114-s006.docx]

**Supplementary Table 1. ORIEN AVATAR Stages II and III CRC patient summary**

|  | | |  |  |  |  |  |  |
| --- | --- | --- | --- | --- | --- | --- | --- | --- |
|  | **Variables** | | **TNM Stage** | | | | | |
|  |  |  | **n (%)** | | | | | |
|  |  |  | **Stage II and III combined** | | **Stage II** | | **Stage III** | |
|  |  |  | 734 (100) | | 325 (44) | | 409 (56) | |
|  | **Gender** | **Male** | 387 (53) | | 180 (55) | | 207 (51) | |
|  |  | **Female** | 347 (47) | | 145 (45) | | 202 (49) | |
|  | **Age (years) at diagnosis** | **Median** | 61 | | 62 | | 60 | |
|  |  | **Range** | 18-86 | | 29-86 | | 18-86 | |
|  | **Primary tumor sidedness** | | **Right** | **Left** | **Right** | **Left** | **Right** | **Left** |
|  |  |  | 343 (47) | 361 (49) | 156 (48) | 158 (49) | 187 (46) | 203 (50) |
|  | **Mutation** | ***KRAS* codon 12, 13 and 61** | 122 (36) | 94 (26) | 47 (30) | 44 (28) | 75 (40) | 50 (25) |
|  |  | ***NRAS* exon** | 13 (4) | 19 (5) | 5 (3) | 13 (8) | 8 (4) | 6 (3) |
|  |  | ***BRAF*** | 60 (17) | 49 (14) | 23 (15) | 24 (15) | 37 (20) | 25 (12) |
|  | **Peri-operative 5-FU** | **Yes** | 116 (34) | 142 (39) | 28 (18) | 37 (23) | 88 (47) | 105 (52) |
|  |  | **No** | 227 (66) | 219 (61) | 128 (82) | 121 (77) | 99 (53) | 98 (48) |
