# Supplementary material for: Proto‐Oncogene HRAS Transcript Level and Overall Survival in Stages II and III Colorectal Cancer
Source: Cancer Med. 2025 Jul 31;14(15):e71114. doi: 10.1002/cam4.71114 (PMC12311480; doi:10.1002/cam4.71114)
Supplement: Supplementary file 14 — Table S2: Cox regression analysis on overall survival of Stages II and III colorectal cancer with high HRAS transcript expression in KRAS(−); BRAF(−); NRAS(−) genotypic background at 5 years. [file CAM4-14-e71114-s008.docx]

**Supplementary Table 2. Cox regression analysis on overall survival of stages II and III colorectal cancer with high *HRAS* transcript expression in *KRAS*(-);*BRAF*(-);*NRAS*(-) genotypic background at 5 years**

| **Variable** | **HR** | **95% CI** | ***p* value** |
| --- | --- | --- | --- |
| Gender (Male vs. Female) | 0.7526 | 0.3053 to 1.833 | 0.5301 |
|  |  |  |  |
| Peri-operative 5-FU | 0.607 | 0.2094 to 1.668 | 0.3398 |
|  |  |  |  |
| Pathological TNM stage (Stage II vs. III) | 0.5353 | 0.1638 to 1.530 | 0.2625 |
|  |  |  |  |
| Tumor sidedness (Left vs. Right) | 3.1 | 1.125 to 10.01 | 0.0386* |
|  |  |  |  |
| Tumor sidedness (Not specified vs. Right) | 3.835 | 0.6943 to 18.88 | 0.0994 |
|  |  |  |  |
| *KRAS* transcript level (High vs. Low) | 2.336 | 1.006 to 5.627 | 0.0505 |
|  |  |  |  |
| *NRAS* transcript level (High vs. Low) | 1.087 | 0.4164 to 3.222 | 0.8706 |
| * *p* value < 0.05 |  |  |  |
